# Gold and silver dichroic nanocomposite in the quest for 3D printing the Lycurgus cup

**DOI:** 10.3762/bjnano.11.2

**Published:** 2020-01-02

**Authors:** Lars Kool, Floris Dekker, Anton Bunschoten, Glen J Smales, Brian R Pauw, Aldrik H Velders, Vittorio Saggiomo

**Affiliations:** 1 Laboratory of BioNanoTechnology, Wageningen University, PO Box 8038, 6700EK, Wageningen, The Netherlands,; 2 Bundesanstalt für Materialforschung und -prüfung (BAM), Unter den Eichen 87, 12205 Berlin, Germany

**Keywords:** 3D printing, dichroism, Lycurgus cup, nanocomposite

## Abstract

The Lycurgus cup is an ancient glass artefact that shows dichroism as it looks green when a white light is reflected on it and a red colouring appears when a white light is transmitted through it. This peculiar dichroic effect is due to silver and gold nanoparticles present in the glass. In this research we show the synthesis of dichroic silver nanoparticles and their embedding in a 3D printable nanocomposite. The addition of gold nanoparticles to the silver nanoparticle composite, gave a 3D printable nanocomposite with the same dichroism effect of the Lycurgus cup.

## Introduction

The Lycurgus cup is, without any doubt, one of the most fascinating glass artefacts in the history of humankind [1]. This 4th century Roman cup, classified as cage cup, is a wonderful masterpiece of glass working from the Roman Empire. Art historians and glass artists alike have wondered at the fabrication of its intricate structure since its first discovery [2]. Whether the cup was used as a drinking cup, lampshade or as decoration is still under debate [3].

The Lycurgus cup is worldwide admired in particular because of its fascinating dichroic property. The cup presents a green colour when the observer and the light source are on the same side (reflection), and a deep red colour when the observer and the light source are at opposite sides (transmission) [4]. This peculiar effect, which has perplexed scientists for centuries, was discovered to be due to the presence of nanoparticles in the glass. This effect was due to two different metallic nanoparticles: silver nanoparticles (AgNP) and gold nanoparticles (AuNP). While the latters are responsible for the red plasmonic colour, the silver nanoparticles cause the green reflection [5–6].

The Lycurgus cup is the only intact ancient glassware exhibiting this optical property. Only a few other small human-made dichroic glass fragments were found around the world [7–8].

## Results and Discussion

We have recently reported the development of a 3D-printable AuNP nanocomposite which presents a dichroic effect, showing a brownish colour in reflection and a violet colour in transmission [9]. Driven by the curiosity of reproducing the green/red dichroic effect of the Lycurgus cup using modern knowledge on nanoparticles and recent technology like 3D printing, we embraced the quest of developing a 3D-printable silver nanocomposite material.

The first step was to synthesise dichroic AgNP. Described in literature, the “Lycurgus effect” can be obtained on surfaces using nanolithography for producing a square lattice which reflects different wavelengths [10]. This fabrication cannot be applied to soft materials, thus could not be 3D printed. In 2014, Tanabe’s group synthesised dichroic AgNP by photochemical reaction using different “white” light sources [11].

The most stunning green/red effect which the group presented was obtained by synthesising AgNP by exposing a solution of the silver precursor, sodium citrate, and a photopolymer initiator to sunlight for four months. When the same starting solution was exposed to incandescent light or xenon light to decrease the synthesis time, the final AgNP solutions presented different colorations. The dichroic effect of the nanoparticles was attributed to their size and their particular dodecahedral shape. Although the green/red dichroism is visually excellent, the four months’ synthesis is a hindrance for fast production and screening of nanocomposites.

**Figure 1:**
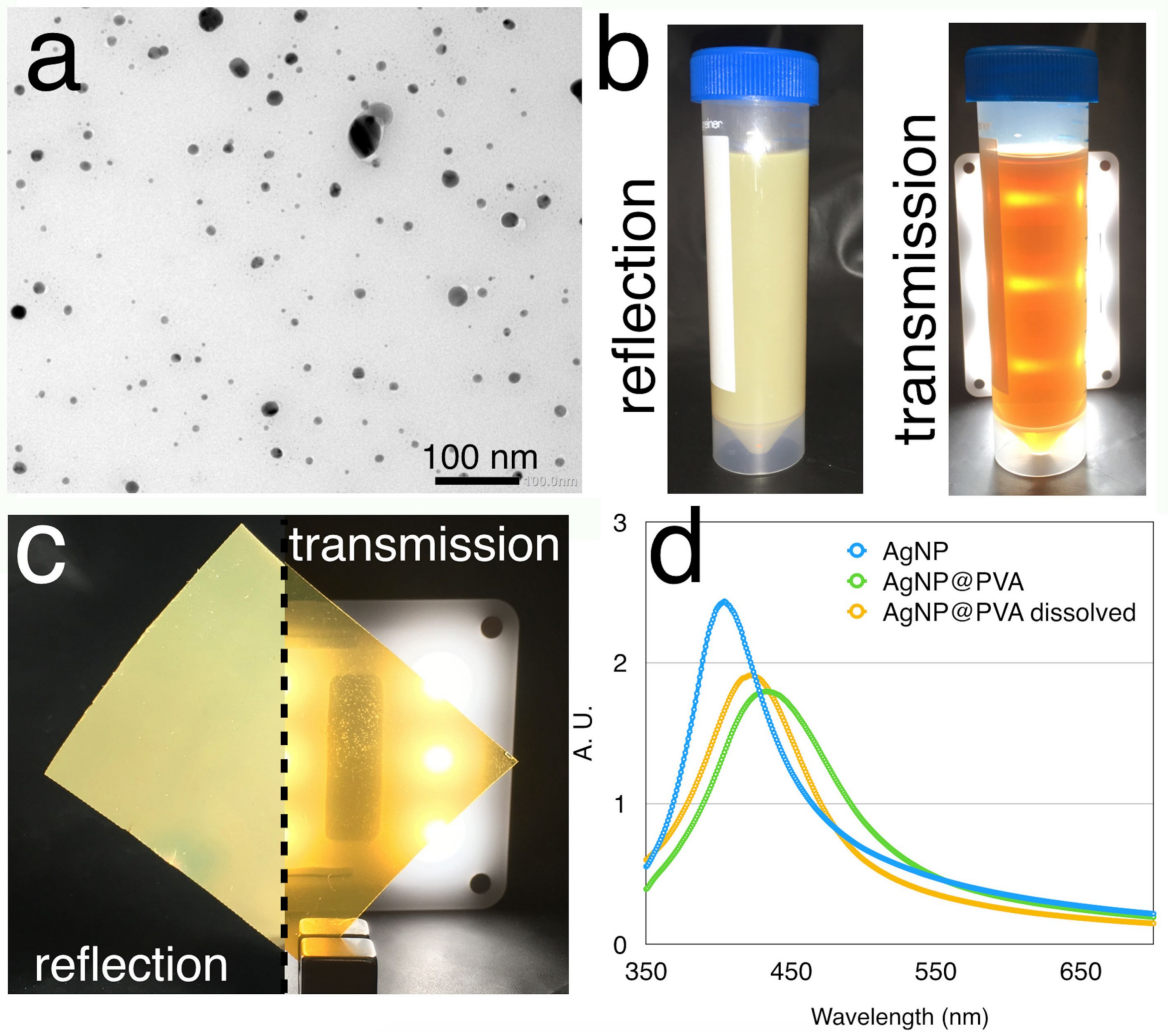
a) TEM picture of the dichroic AgNP solution; b) the AgNP solution shows the dichroic effect presenting a greenish colour when illuminated from the front and a yellowish/orange colour when illuminated from behind. c) when the nanoparticles are embedded in PVA, the nanocomposite material shows the same dichroic effect of the bulk solution. d) UV–vis spectra of the AgNP in solution (blue), embedded in PVA (green) and after the AgNP@PVA has been redissolved in water (yellow).

Our previously reported dichroic AuNP were synthesised using a sub-stochiometric ratio of reducing agent compared to a standard Turkevich method. The smaller amount of reducing agent resulted in bigger ellipsoidal AuNP which presented a dichroic effect. Using the same approach, a low amount of reducing agent, for the synthesis of AgNP did not yield the hoped dichroic effect, and the nanoparticles precipitated out of solution as a black solid. A low amount of reducing agent in the presence of polyvinyl alcohol (PVA) as stabiliser agent, which would be the 3D printable material, also did not suffice to keep the nanoparticles in solution. After multiple variations, we found that reducing silver ions at room temperature immediately followed by an addition of a polyvinylpyrrolidone (PVP) solution formed dichroic silver nanoparticles in minutes. The addition of the reducing agent (NaBH_4_) to a silver nitrate solution forms nanoclusters, and the immediate addition of PVP stabilises those nanoparticles, quenching the reduction while the nanoparticles are still of the right dimensions to be dichroic. If the lag between the reduction step and the addition of the PVP is too long, i.e., more than one minute, then the NaBH_4_ reduces all the silver to small, yellow nanoparticles which are not dichroic. TEM analyses showed that the mixture is polydisperse ranging from small nanoparticles of less than 3 nm to a population of nanoparticles in the range of 30 nm (Figure 1a and Supporting Information File 1, Figures S1 and S2). The AgNP solution is dichroic and when illuminated from the front it shows a greenish colour (reflection), while illumination from behind shows a more yellowish/orange colour (reflection and transmission) (Figure 1b). We speculate that the yellow colour is due to the plasmonic colour of the small particle sizes, while the green colour is due to the bigger particles which backscatter more. Backscattering is a function of the fourth power of the radius of the particles and even minute amount of large particles may be significant in reflecting light [12].

We embedded the solution containing the dichroic AgNP in PVA, firstly by dissolving the PVA in water and adding the AgNP solution to reach a ratio of 0.2% w/w of Ag in PVA, and then evaporating the water in a 70 °C oven until the plastic material was dried. The AgNP@PVA presents the same dichroic effect of the AgNP solution, showing a greenish reflection and yellowish transmission (Figure 1c). The composition of nanoparticles in solution and in PVA was studied by UV–vis. The AgNP solution presents a peak around 410 nm, in accordance with small yellow spherical silver nanoparticles, and a long absorption tail because of the polydispersity. When embedded in PVA, the peak shows a redshift of about 30 nm. When the AgNP@PVA is dissolved in water, the peak did not come back to its exact original wavelength but it retains a small redshift (Figure 1c). Those effects can be explained by the environment of the silver nanoparticles in solid PVA and by the presence of PVA after the dissolution of AgNP@PVA which can compete with PVP on the nanoparticle surface. We studied this effect also by SAXS, comparing the size distribution of the nanoparticles in solution, the ones from the dissolved AgNP@PVA and also the ones in a 3D printed AgNP@PVA. SAXS analyses of the three samples do not show any great differences in the size distribution of the nanoparticles (Supporting Information File 1, Figure S3), proving that the nanoparticles do not show drastic changes during the fabrication and the 3D printing of the AgNP@PVA.

**Figure 2:**
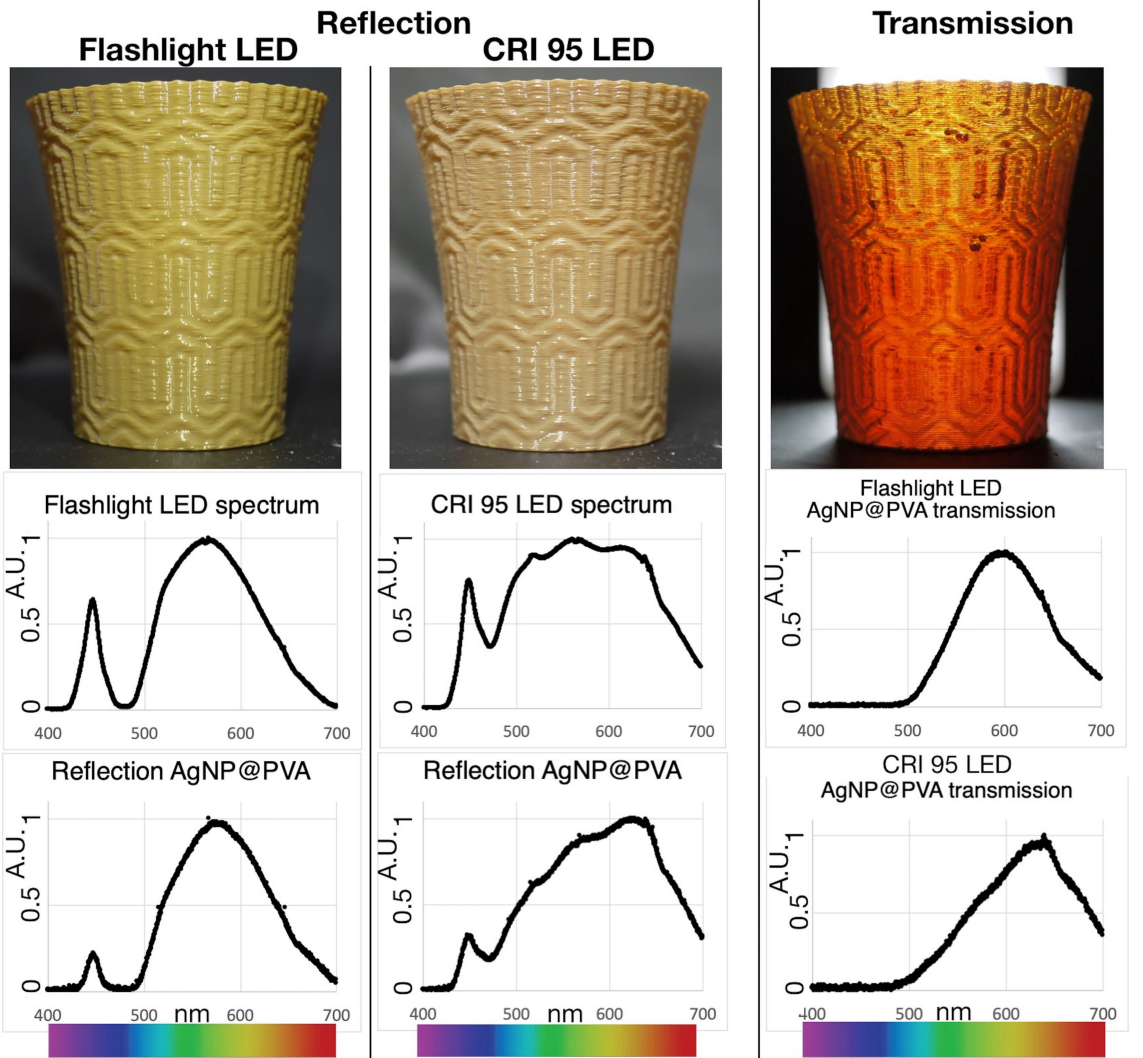
3D printed AgNP@PVA cup under reflection and transmission. When the cup is illuminated by a flashlight LED it reflects a greenish colour as evident from the reflectance spectrum. However, when illuminated by a broader coverage white light as the CRI 95 LED, it reflects mostly near the red region of the spectrum. In transmission, the nanocomposite shows a red coloration, independently by the illumination source.

We extruded the nanocomposite into a 3 mm filament which was used to print a cup using a standard, off-the-shelf fused deposition model (FDM) 3D printer. The nanocomposite showed the same printability of the non-augmented material. The 3D printed cup presented a greenish colour when illuminated from the front and an orange colour when illuminated from the back, a similar behaviour of the Lycurgus cup (Figure 2a and the video in Supporting Information File 2). Moreover, we noticed another particular behaviour. When the 3D printed cup was illuminated from the front by a smartphone flashlight, the perceived colour was green, but when, using the same angle of illumination, it was illuminated by a “solar-like” LED array, the cup reflection shifted to a more brownish colour (Figure 2 and video in Supporting Information File 3). The difference between the two light sources lies in the coverage of the visible spectra. Even though the flashlight is perceived as white light, its spectrum lacks of red coverage while the “solar-like” LEDs have a broader coverage of the visible spectrum. The “solar-like” LEDs have a colour rendering index (CRI), which is a standard used for illumination sources, of more than 95, where a CRI of 100 is classified as the illumination from a black body. In the rest of this article, we will refer to “flashlight LED” and “CRI 95 LED” for specifying the two different light sources. In the sunlight, the cup also reflects light with a brownish colour, similar to the CRI 95 LED illumination (Supporting Information File 1, Figure S4). Although this behaviour surprised us in the beginning, it is obvious that scattering and reflection are highly influenced by the light source, meaning that an object can scatter and reflect only wavelengths that are present in the light source. If the light source lacks, for example, strong intensities in the red region of the spectrum, the illuminated object cannot scatter or reflect such wavelengths. The transmitted colour does not show this drastic change and it remains orange. In certain ways, this effect is also evident in the real Lycurgus cup, which shows a brownish colour when in ambient light, and the green reflected colour only when a picture is taken using a flash (Supporting Information File 1, Figure S5). However, cameras have automatic white-balancing and colour corrections and therefore prone not to show the real colour but an algorithm enhanced picture. To have a more reliable and scientific method to study the reflected and transmitted colours we measured the 3D printed material using a reflectance spectrophotometer with the two different light sources (Figure 2). From the spectra of the two light sources, it is clear that the CRI 95 LED covers a larger part of the visible spectrum compared to the flashlight LED. The reflectance spectra of the 3D printed AgNP@PVA reflect this discrepancy in the illumination system, showing a greener colour when illuminated by the flashlight LED and a red-shifted spectrum when illuminated by the CRI 95 LED. The transmission spectra are more similar for the two illumination systems, with a redder coloration when illuminated by the CRI 95 LED.

**Figure 3:**
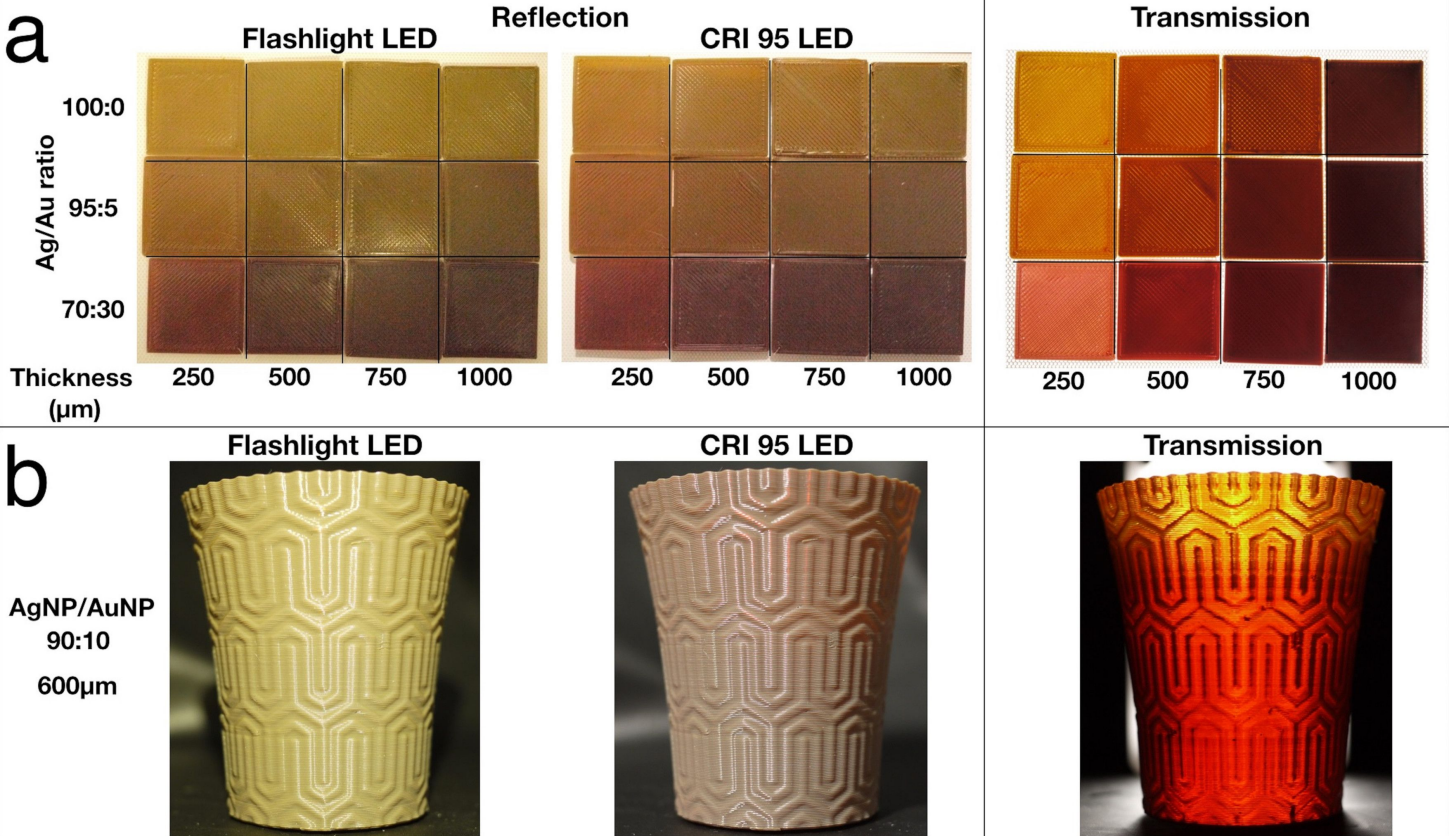
a) 3D printed square specimens using a different ratio of Ag/Au and various thicknesses. In reflection, the flashlight LED causes a more greenish colour reflection, while the CRI 95 LED shows a more brownish reflection. The brown reflection increases when increasing the amount of AuNP in the nanocomposite, this is due probably to the absorption of the nanoparticles. In transmission, the thicker the material, the redder the colour transmitted. b) A 3D printed cup using a ratio of Ag/Au of 90:10 and a thickness of 600 µm under different illumination systems, which shows the same dichroic effect of the Lycurgus cup.

Although the reflected and transmitted colours of the 3D printed AgNP nanocomposite are already similar to the original Lycurgus cup, the latter is also composed of gold nanoparticles, which render the transmitted light redder than orange. We therefore, embarked on the fabrication of the mixed gold and silver nanocomposite. It should be noted that the gold nanoparticles in the Lycurgus cup are partially affecting the green reflected colour, and their effect is more evident in the red transmitted one, thus, the gold nanoparticles themselves are not dichroic. We synthesised 16–20 nm spherical gold nanoparticles with a standard Turkevich method (Supporting Information File 1, Figure S6). Mixing the two solutions of gold and silver nanoparticles and then embedding them together in PVA did not result in the desired effect but in a brown coloured solution. We speculate that, as both nanoparticles are not coated with strong ligands, they are prone to metallic exchange between them. To avoid this, we embedded gold and silver nanoparticles separately in PVA for manufacturing AuNP@PVA and Ag@PVA nanocomposites (Supporting Information File 1, Figure S7). Those two materials were then extruded together in different ratios for making 3D printable filament. We printed different specimens using different percentages of AuNP and different thicknesses (Figure 3a). It is clear that, at a higher percentage of gold nanoparticles, the material tends to reflect a brown colour, with a more greenish shade when illuminated by the flashlight LED. The transmitted light is, obviously, affected also by the amount of light passing through it, thus, the bigger the thickness, the darker the colour. Also, in this case, the presence of gold nanoparticles in the material shows a redshift compared to the AgNP@PVA, which in small layers tends to show a more yellowish coloration (Supporting Information File 1, Figure S8).

**Figure 4:**
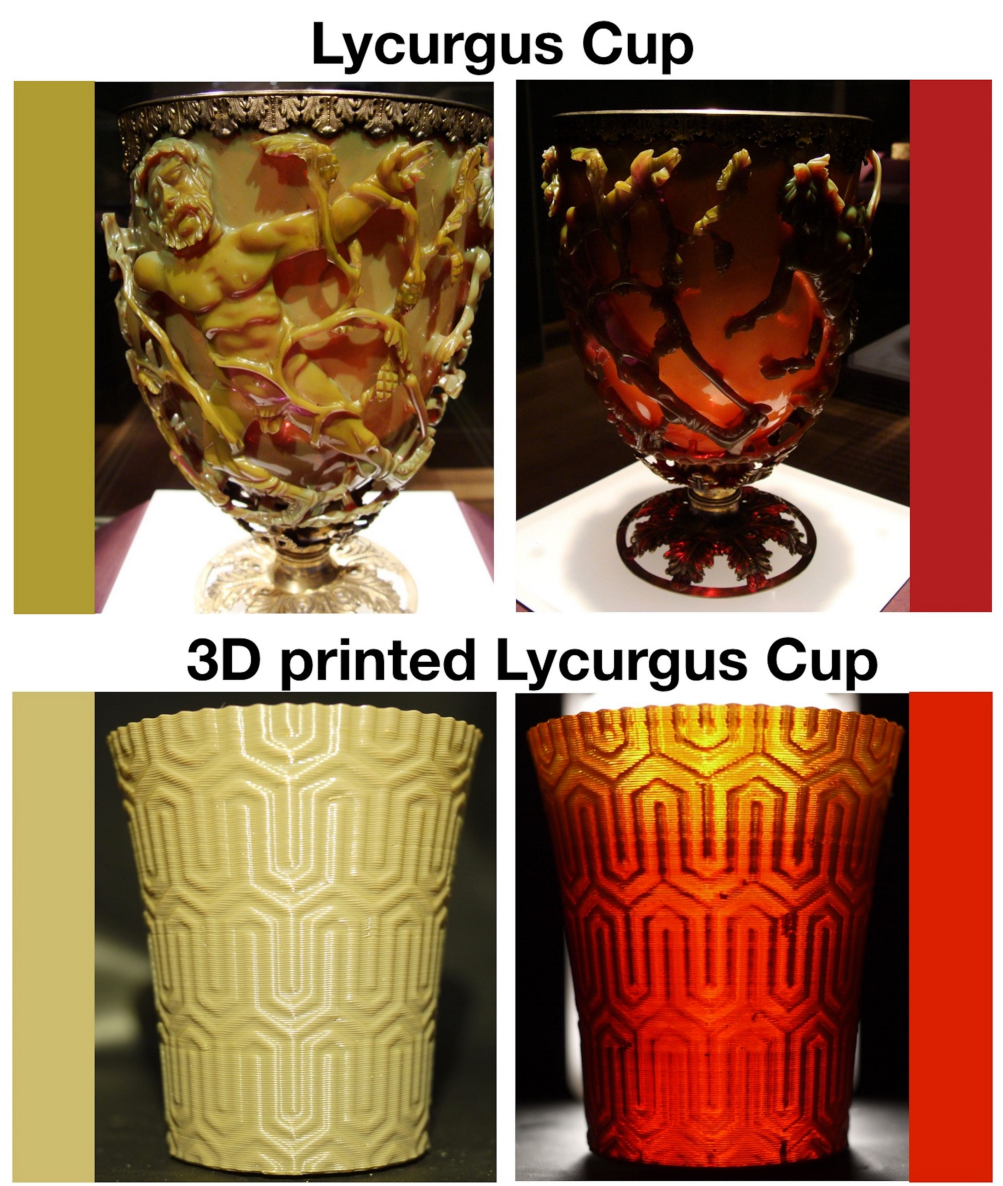
Comparison between the original Lycurgus cup and the 3D printed Ag/Au@PVA nanocomposite cup presented in this research using the flashlight LED as the light source. The boxed colours represent the majority of the colour of the cups in reflection and in transmission. Pictures of the Lycurgus cup provided by the courtesy of Lucas Livingston (ancientartpodcast.org), Copyright (2013) Lucas Livingston (ancientartpodcast.org), published under CC BY 2.0 (https://creativecommons.org/licenses/by/2.0/).

After this test, we produced a mixed nanocomposite filament of AgNP containing 10% of AuNP which would produce the most similar effect to the original Lycurgus cup. We printed a cup using a 600 µm thickness and examined the cup under different illumination. The cup shows the classical green/red colour shift when the incident light is shone from the front of the object or from behind (Figure 3b). This is even more impressive when the 3D printed cup is presented together with pictures from the Lycurgus cup where the similarity is striking (Figure 4). The matching colours are still not perfect, as the green and the red colour of the 3D printed cup are duller compared to the Lycurgus cup. Obtaining the identical colours of the Lycurgus cup might be possible by studying the reflectance and transmittance spectra from the Lycurgus cup and matching them with freshly synthesised Au and Ag nanoparticles in PVA. However, such spectra are missing in literature and the only few present are hand-drawn [13], making it impossible to properly tune and match the colour, but only mix and match for a hit or miss experiments. We have nevertheless proven that it is possible to create the Lycurgus effect in 3D printed material.

## Conclusion

This research started as curiosity-driven research: can we, using modern knowledge about plasmonic metallic nanoparticles and modern technology such as 3D printing, recreate an artefact from more than 1600 years ago which has puzzled scientists for centuries?

The results presented here are more general and can be applied in chemistry, physics and materials science. For example, the colour of the materials is not coming from organic dyes or inorganic complexes but from the plasmonic effect of nanoparticles, which are more stable to photobleaching or degradation. Using the methodology presented here it is also possible to synthesise plasmonic nanocomposite 3D printable smart materials, which behave differently to different angles of illumination.

## Experimental

### General

Silver nitrate (Sigma-Aldrich), sodium borohydride (Sigma-Aldrich), polyvinylpyrrolidone K30 (MW 40 KDa, Alfa Aesar), chloroauric acid trihydrate (Alfa Aesar), trisodium citrate dihydrate (Sigma-Aldrich) were purchased and used without further purification. PVA as 3D printable filament (ultimaker) was bought from a local vendor (https://makerpoint.nl). For extruding the printable filament a Felfil Evo (https://felfil.com) was used and the filament was printed using a Ultimaker 2+ (Ultimaker) FDM printer.

A Shimadzu UV1601 UV–vis was used for the UV–vis study. TEM samples were imaged in a JEOL 1400Plus TEM operating at 120 kV.

SAXS: Long-range ordering was studied by small angle X-ray scattering (SAXS) in Multiscale Analyser for Ultrafine Structures (MAUS): a heavily customized Xeuss 2.0 (Xenocs, France). X-rays are generated from a microfocus X-ray tube with a copper target, followed by a multilayer optic to parallelize and monochromatize the X-ray beam to a wavelength of 0.154 nm. The detector consists of an in-vacuum motorized Eiger 1M, placed for this investigation at distances of 208, 1258 and 2508 mm from the sample. The space between the start of the collimation until the detector is a continuous, uninterrupted vacuum to reduce background. Solid samples were fixed in place, within the evacuated sample chamber, whilst liquid samples were flowed through a low-noise cell equipped with SiN windows. The resulting data has been processed and scaled to absolute units using the DAWN software package according to standardized procedures [14–15].

Reflectance spectra were obtaining by using the flashlight LED and the CRI 95 LED as light sources at an angle of about 60°, and a thorlab ccs200/m compact spectrometer with a fiber optic as detector. Spectra were normalized against the maximum intensity.

The flashlight LED used was the LED light of an iPhone SE, and the CRI 95 LED was an Aputure AL-M9. The pictures were recorded using a Panasonic Lumix DMC-GF2.

### Synthesis of dichroic AgNP

Silver nitrate (190 mg, 1.1 mmol) of was dissolved in 150 mL of DI water in a 500 mL Erlenmeyer flask at room temperature. Under vigorous stirring, 20 mg (0.5 mmol) of sodium borohydride in 30 mL of DI water were added in one shot quickly followed by the addition of 1.5 g of PVP dissolved in 60 mL of DI water (in one shot). The solution became dichroic after few minutes. For obtaining dichroic AgNP it is important that the sodium borohydride and the PVP are added in quick succession (less than 30 seconds) for freezing the reduction of the bigger nanoparticles. If the PVP is added in a later stage, only small AgNP will be produced, ending in a yellow transparent solution.

### Synthesis of AuNP

The AuNP were synthesized using the Standard Turkevich method [16]. In brief, 95 mL of 0.14 mM gold precursor solution (0.133 mL of 0.1 M HAuCl_4_ in 95 mL of DI water) was heated to 100 °C. The reduction of the gold precursor solution was initiated by adding 5 mL of 34 mM citrate solution (50 mg of citrate), while stirring vigorously. After a few minutes, the mixture turned grey followed by a gradual color change to red over a period of about 10 minutes.

Preparation of Au@PVA and Ag@PVA nanocomposite 3D printable filaments: The procedure for preparing the 3D printable nanocomposite is similar to the procedure described in [9].

3D-printable PVA filament was dissolved in water to reach a concentration of 20% w/w of PVA in water. Once the PVA was dissolved, the solution of AuNP or AgNP were added to reach a concentration of 0.2% w/w Ag/PVA and 0.1% w/w Au/PVA. The mixture was placed in large containers and left in an oven at 70 °C overnight, until all the water evaporated.

When the plastic was completely dried, it was shredded with the aid of a kitchen blender (Tristar BL-4445) and passed through a 3D printed sieves with holes of 3 mm. The resulting pellets were extruded to a filament of 2.85 mm using a Felfil Evo at 190 °C and rotation speed of 7. The material was stored in a sealed plastic bag with desiccant for avoiding moisture.

### 3D printing

3D design of the cup was obtained via https://thingiverse.com from the user “joris” https://www.thingiverse.com/thing:147512 and used under Creative Commons CC-By-NC 3.0.

The 3D designs were sliced with Cura 3.1.0 and printed on a Ultimaker 2+ FDM printer using a 0.6 mm nozzle (0.25 mm layer height and 0.6 mm wall thickness). Printing parameters used were: Printing temperature 215 °C, build plate temperature 60 °C, fan speed 50%, print speed 50 mm/s.

## Supporting Information

File 1 TEM micrographs of Ag and Au nanoparticles, SAXS data, pictures of the Lycurgus cup under different illumination, transmission and reflectance spectra of AuNP/AgNP @PVA nanocomposites.

File 2 Video of dichroic Ag nanoparticles and 3D printed nanocomposites.

File 3 Video of dichroic 3D printed nanocomposites under different illumination systems.
