# Supplementary material for: Gold and silver dichroic nanocomposite in the quest for 3D printing the Lycurgus cup
Source: Beilstein J Nanotechnol. 2020 Jan 2;11:16–23. doi: 10.3762/bjnano.11.2 (PMC6964654; doi:10.3762/bjnano.11.2)
Supplement: File 1 — TEM micrographs of Ag and Au nanoparticles, SAXS data, pictures of the Lycurgus cup under different illumination, transmission and reflectance spectra of AuNP/AgNP @PVA nanocomposites. [file Beilstein_J_Nanotechnol-11-16-s001.pdf]

BEILSTEIN JOURNAL OF NANOTECHNOLOGY

Supporting Information

for

Gold and silver dichroic nanocomposite in the quest for 3D printing the Lycurgus cup

Lars Kool, Floris Dekker, Anton Bunschoten, Glen J. Smales, Brian R. Pauw,
Aldrik H. Velders and Vittorio Saggiomo

Beilstein J. Nanotechnol. **2020,** *11,* 16–23. doi:10.3762/bjnano.11.2

TEM micrographs of Ag and Au nanoparticles, SAXS data, pictures of the Lycurgus cup under different illumination, transmission and reflectance spectra of AuNP/AgNP @PVA nanocomposites

License and Terms: This is a supporting information file under the terms of the Creative Commons Attribution License (https://creativecommons.org/licenses/by/4.0). Please note that the reuse, redistribution and reproduction in particular requires that the authors and source are credited.
The license is subject to the *Beilstein Journal of Nanotechnology* terms and conditions: (https://www.beilstein-journals.org/bjnano)

Table of contents **Page**

Table of contents	Page
Figure S1: TEM micrographs of dichroic AgNP	S1
Figure S2: TEM micrographs before and after dissolving AgNP@PVA	S2
Figure S3: SAXS data	S2
Figure S4: Sunlight pics	S3
Figure S5: Lycurgus flash	S3
Figure S6: AuNP TEM	S4
Figure S7: AuNP AgNP @PVA	S4
Figure S8: Spectra transmission reflection AuNP/AgNP @PVA material	S5

Supporting Information File 2: Video about the dichroic AgNP@PVA and 3D printed cups (Music: Gigue From 3rd Cello Suite, Exzel Music Publishing (freemusicpublicdomain.com) Licensed under Creative Commons by Attribution 3.0 (CC-BY 3.0)

Supporting Information File 2: Video S2 AgNP@PVA 3D printed cup under different illumination systems.

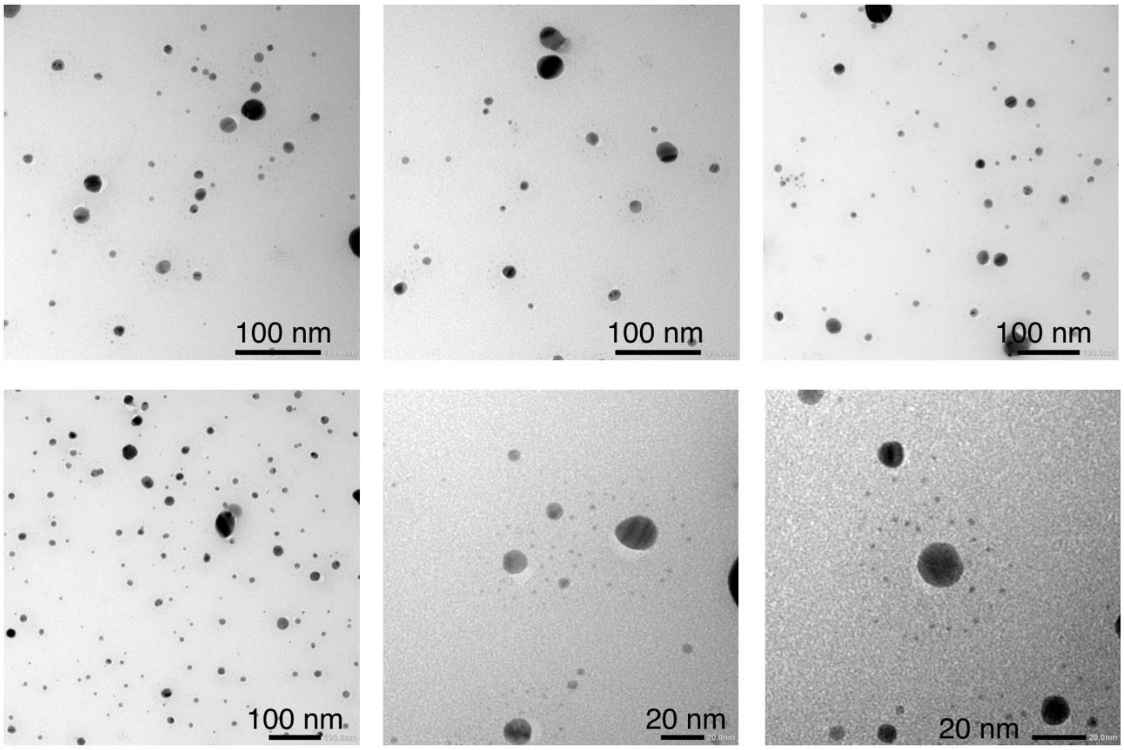

Figure S1. TEM pictures of the dichroic AgNP.

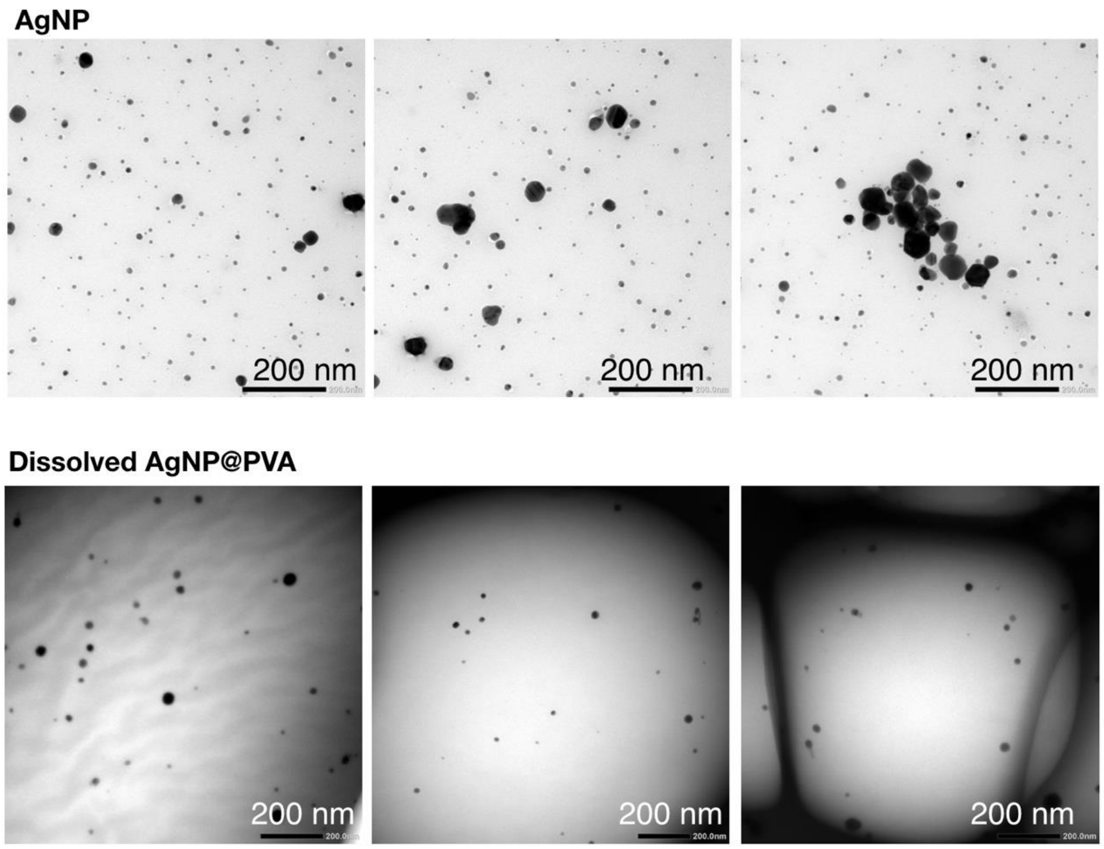

Figure S2. TEM micrographs of dichroic AgNP solution (top), and after dissolving the AgNP@PVA in water (bottom).

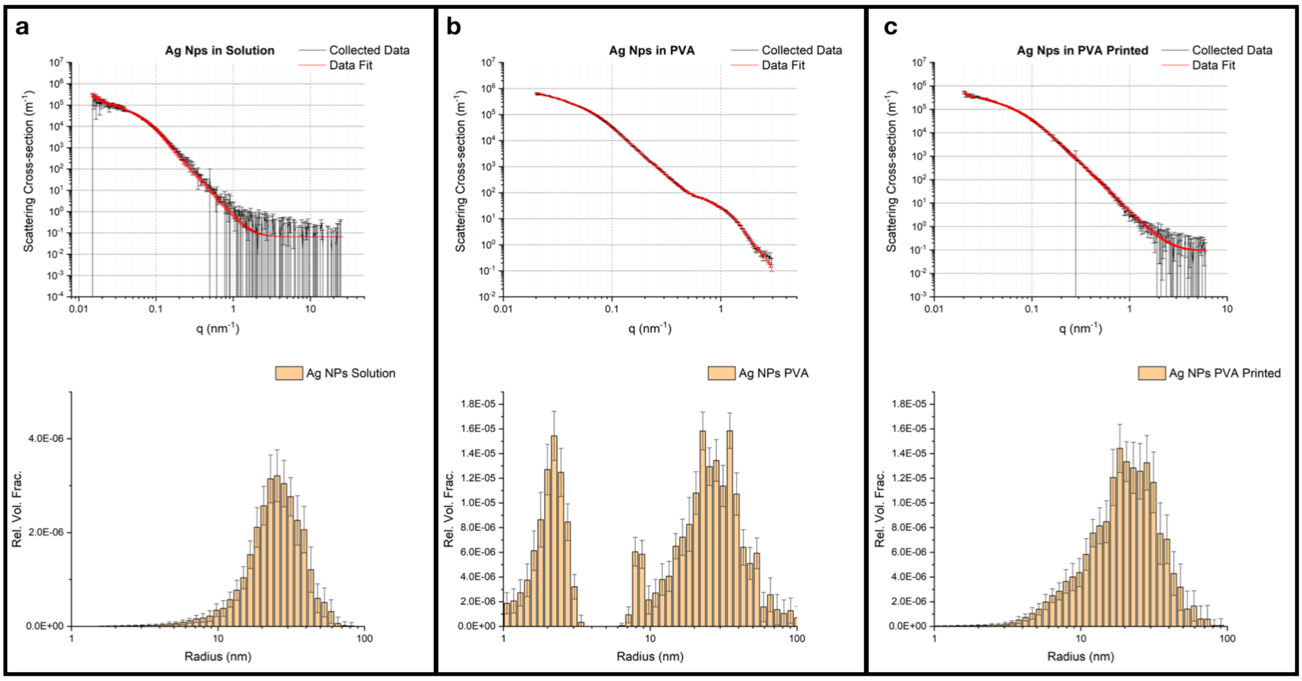

Figure S3. SAXS of a) AgNP in solution, b) AgNP@PVA and c) 3D printed AgNP@PVA. We speculate that the smaller particles detected in the AgNP@PVA sample are due to the phase separation of PVP in PVA. When the solution of AgNP, which contains an excess of PVP is mixed with the PVA solution, the temperature of evaporation for making the solid plastic, is only 70 °C, which is way lower than its melting temperature. Once the material is printed at 215 °C (above the PVP melting point), the two polymers mixes and the smaller PVP particles are no longer detectable.

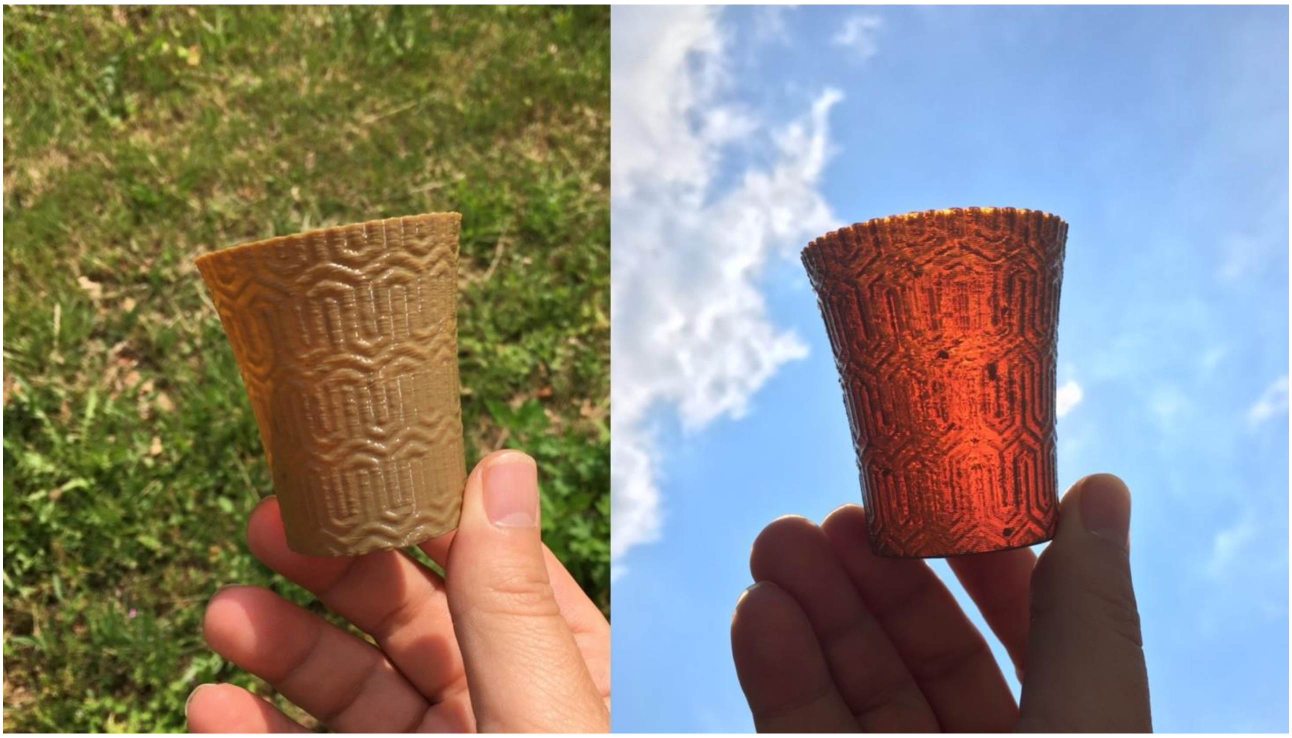

Figure S4. The 3D printed AgNP@PVA dichroic material under sunlight, reflected light on the left and transmitted light on the right.

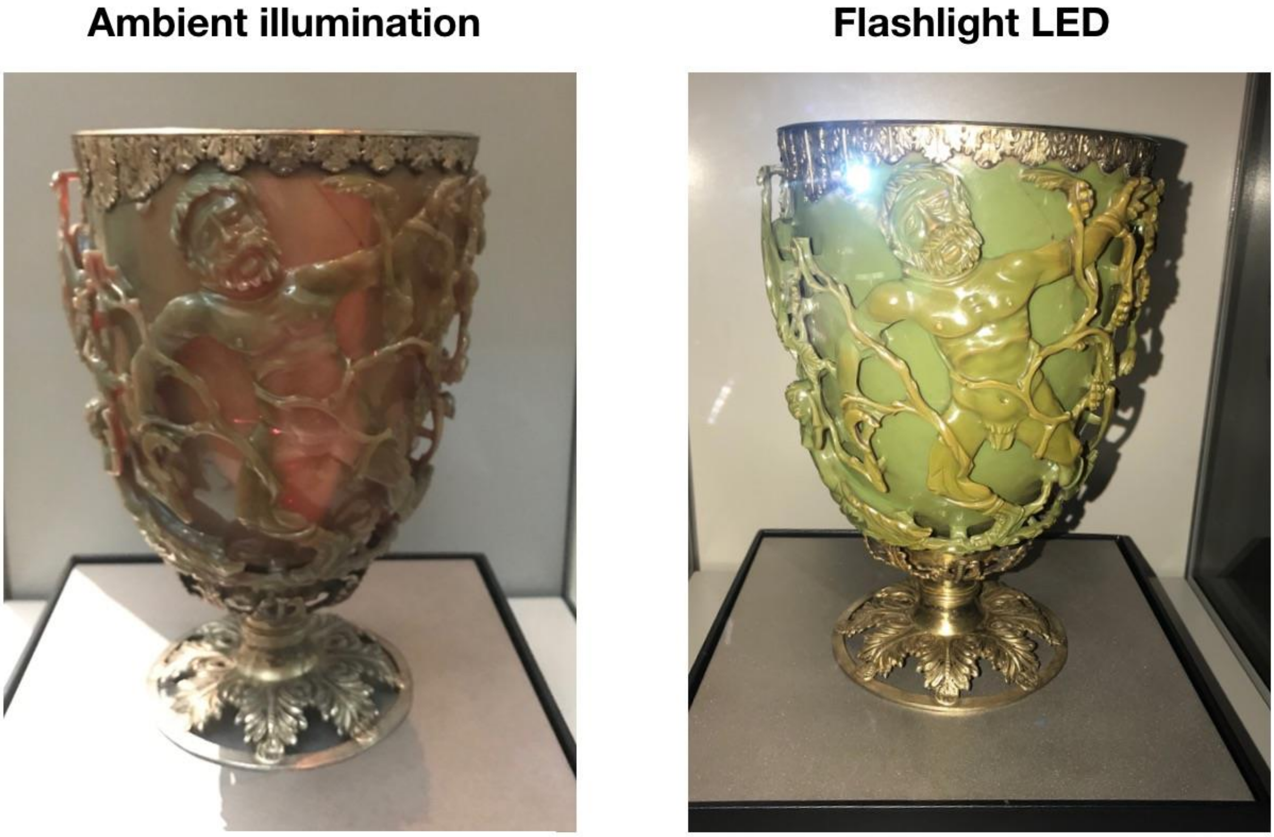

Figure S5. Picture of the Lycurgus Cup under ambient (museum) illumination on the left, and picture taken with the iPhone 7 flash on the right. The flashlight clearly reflects a green colour with respect to the ambient light which makes the cup reflect a more brownish colour. Pictures taken and used with permission from Kirsty Milligan (Twitter: @MilliganKirsty1)

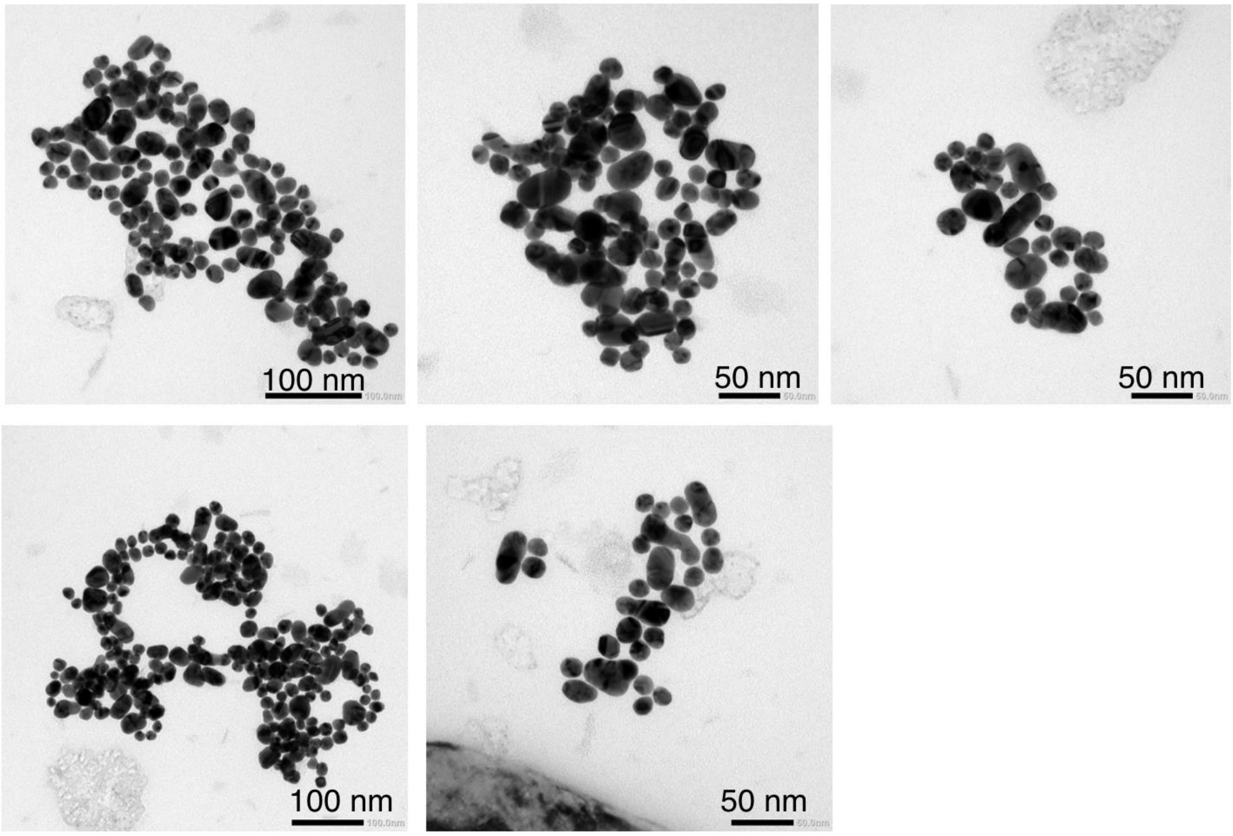

Figure S6. TEM micrographs and particle analyses of the AuNP solution. The AuNP have a peak distribution around 16-20 nm and are mostly spherical (ratio 1.1).

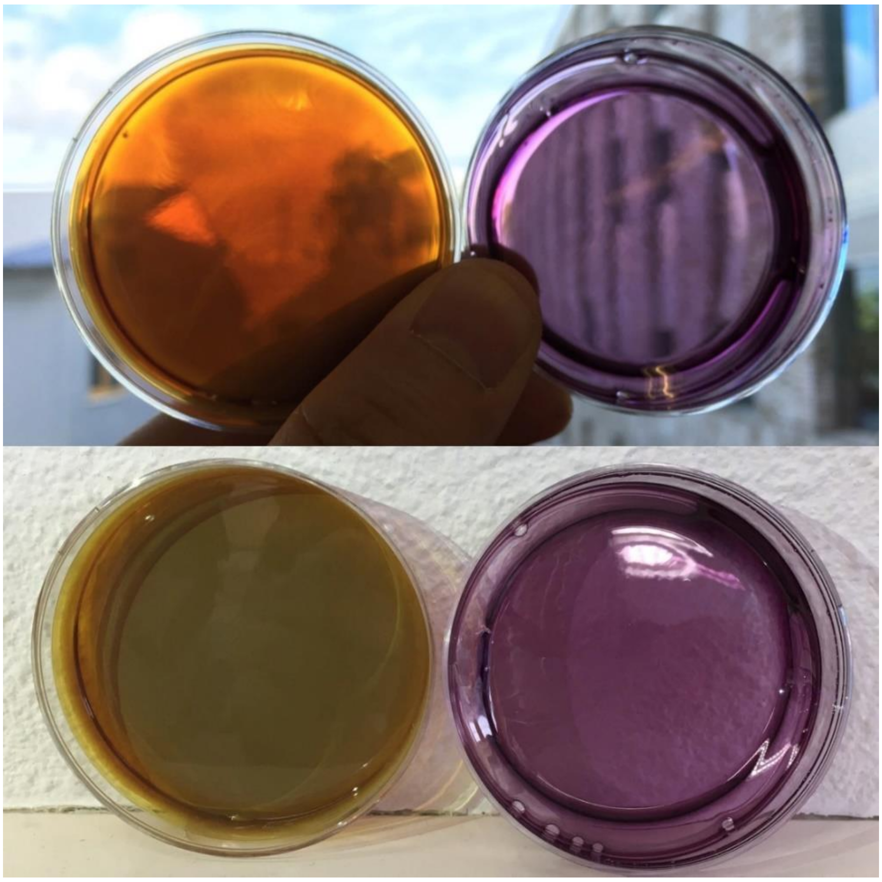

Figure S7. Dichroic AgNP@PVA on the left and AuNP@PVA on the right. Transmitted light on top and reflected light on the bottom.

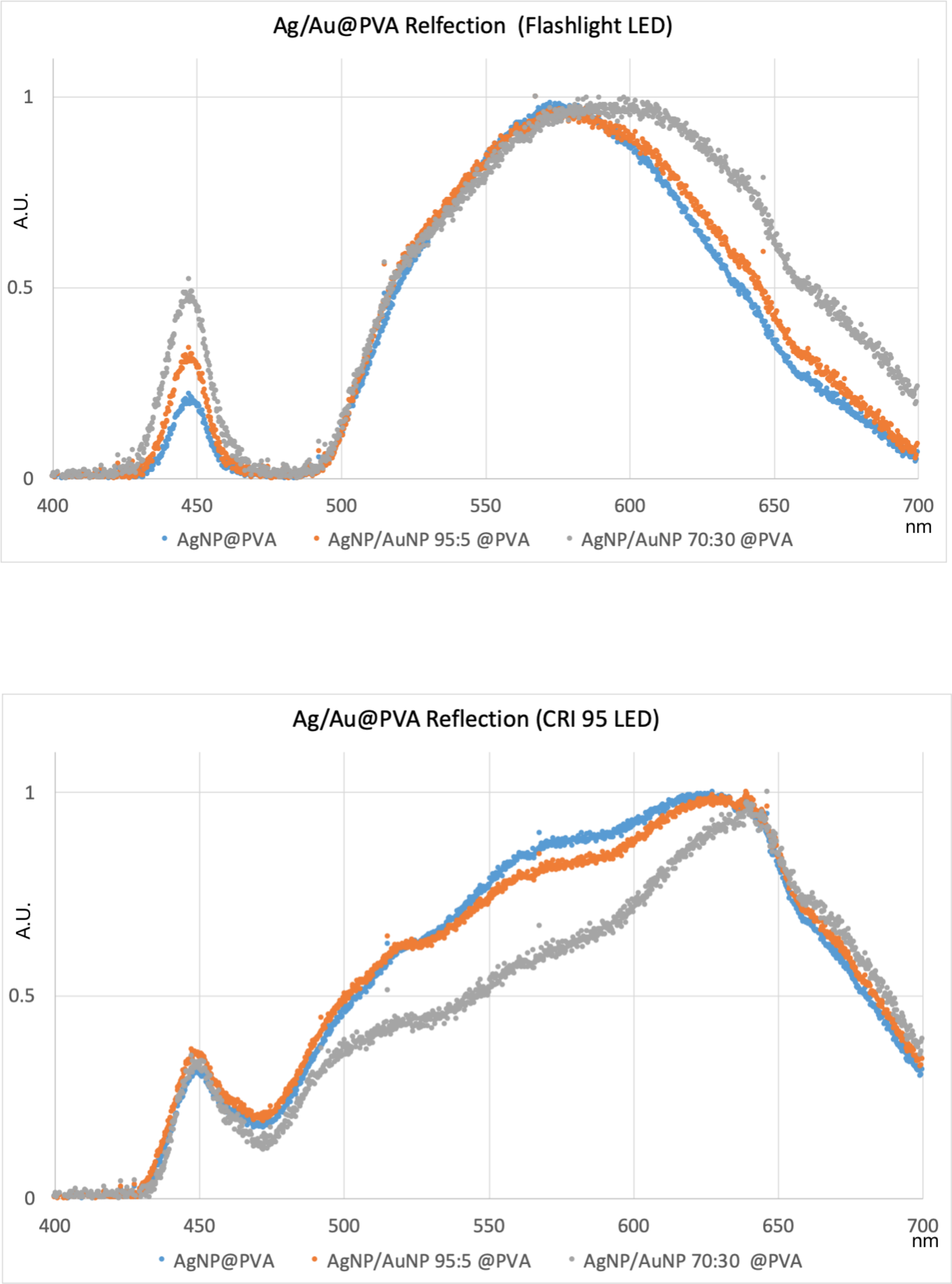

Figure S8. Reflectance spectra of 3D printer mixed AgNP/AuNP @PVA materials. In both illumination cases the reflected light shift to red with the increasing of the AuNP in the material.
